# Child Pain Intensity and Parental Attitudes toward Complementary and Alternative Medicine Predict Post-Tonsillectomy Analgesic Use

**DOI:** 10.3390/children7110236

**Published:** 2020-11-19

**Authors:** Jaclyn Lee, Katherine Delaney, Molly Napier, Elizabeth Card, Brittany Lipscomb, Jay Werkhaven, Amy S. Whigham, Andrew D. Franklin, Stephen Bruehl, Amanda L. Stone

**Affiliations:** 1 Vanderbilt University School of Medicine, Nashville, TN 37212, USA; jaclyn.lee@vanderbilt.edu; 2 Monroe Carell Jr. Children’s Hospital at Vanderbilt, Vanderbilt University Medical Center, Nashville, TN 37212, USA; katherine.e.grierson@vumc.org (K.D.); molly.j.napier@vumc.org (M.N.); 3 Executive Nursing Administration, Nursing Research Office, Vanderbilt University Medical Center, Nashville, TN 37212, USA; elizabeth.b.card@vumc.org; 4 Department of Otolaryngology-Head and Neck Surgery, Vanderbilt University Medical Center, Nashville, TN 37212, USA; brittany.lipscomb@vumc.org (B.L.); jay.werkhaven@vumc.org (J.W.); amy.s.whigham@vumc.org (A.S.W.); 5 Surgical Outcomes Center for Kids, Vanderbilt University Medical Center, Nashville, TN 37212, USA; 6 Department of Anesthesiology, Vanderbilt University Medical Center, Nashville, TN 37212, USA; andrew.franklin@vumc.org (A.D.F.); stephen.bruehl@vumc.org (S.B.)

**Keywords:** children, oxycodone, pediatric pain, adenotonsillectomy, otolaryngology, opioids

## Abstract

Parental attitudes regarding pain interventions and perceptions of their child’s pain intensity likely influence the decision to administer postoperative analgesics. Our study examined the impact of daily fluctuations in child pain intensity and parental attitudes regarding complementary and alternative medicine (CAM) on analgesic administration following pediatric tonsillectomy. Parents of children undergoing tonsillectomy (n = 33) completed a survey assessing CAM attitudes and a 7-day postoperative electronic daily diary to record their child’s daily pain intensity and analgesic medications (acetaminophen, ibuprofen, or oxycodone). Generalized linear mixed models with Poisson distributions evaluated the effects of within-person (child’s daily pain intensity) and between-person (average postoperative pain, parental CAM attitudes) components on the number of medication doses administered. Higher daily pain intensity was associated with more oxycodone doses administered on a given day, but not acetaminophen or ibuprofen. Positive parental CAM attitudes were associated with less oxycodone use, beyond the variations accounted for by the child’s daily pain intensity and average postoperative pain. Both parental CAM attitudes and their child’s daily pain intensity were independently associated with parental decisions to administer opioids following tonsillectomy. Understanding factors influencing individual variability in analgesic use could help optimize children’s postoperative pain management.

## 1. Introduction

Tonsillectomies are one of the most-frequently-performed surgical procedures in the United States, with over 500,000 pediatric cases each year, and often in combination with an adenoidectomy [1,2]. The most common indications for surgery are recurrent tonsillitis and sleep-disordered breathing, especially obstructive sleep apnea (OSA) [3]. Post-tonsillectomy pain is most severe during the first three days following surgery and may be significant for a week or longer [4]. Severe pain is a major cause of post-tonsillectomy morbidity, leading to diminished oral intake, dehydration, and weight loss [5,6,7].

Without explicit guidelines for post-tonsillectomy pain management, variations exist in clinical practice [5,6]. Frequently-used medications include over the counter (OTC) analgesics (typically acetaminophen and ibuprofen) and opioids (typically hydrocodone and oxycodone) [8]. The risks of opioids are well known, including sedation (particularly concerning for OSA patients), constipation, pruritis, and nausea [9,10]. Meanwhile, complementary and alternative medicine (CAM) pain management strategies have grown in popularity as non-pharmacologic treatment options, with pediatric interventions including sound, aromatherapy, honey, and acupuncture [11]. Studies have found parent education and coaching in behavioral strategies such as distraction can help reduce postoperative pain [12,13].

A major influence on pediatric pain management strategies are the beliefs and behavioral tendencies of caregivers [14,15]. Parent concerns regarding side effects of pain medications have been associated with greater use of non-pharmacological techniques to manage children’s pain [16]. On the other hand, parents who favor non-pharmacological techniques may give fewer analgesic doses, especially opioids, following surgery.

This study sought to evaluate the relationship between pediatric pain intensity and parental administration of acetaminophen, ibuprofen, and opioid analgesics in the first seven days following tonsillectomy. We hypothesized that opioid administration would be significantly related to a child’s daily pain intensity, as opioids are prescribed for breakthrough pain, while a weaker relationship was anticipated between pain and OTC analgesics that are typically given on a schedule. Further, we aimed to evaluate the extent to which parental CAM attitudes could predict the number of medication doses administered, beyond the influence of child pain intensity, with the hypothesis that more positive CAM attitudes would lead to fewer doses of administered analgesics.

## 2. Materials and Methods

This was a single-institution prospective study approved by the Vanderbilt University Medical Center (VUMC)’s Institutional Review Board (IRB#: 190112). Participants were drawn from a larger study of pediatric patients aged 2 to 17 years undergoing tonsillectomies at Monroe Carell Jr. Children’s Hospital at Vanderbilt from January to December 2019. The current study focused on a subset of these participants, aged 5 to 17 years who received an opioid prescription upon discharge postoperatively. 

### 2.1. Study Population 

Participants were recruited on the morning of surgery and enrolled after providing parental consent and child assent. Study data were collected and managed using REDCap (Research Electronic Data Capture), a secure, web-based software platform designed to support data capture for research studies, hosted at Vanderbilt University [17,18]. Prospective data collection for one week post-tonsillectomy utilized REDCap-based electronic surveys, sent via email or text message, completed daily by parents. Exclusion criteria included additional contemporaneous surgery with pain implications, significant developmental delay or genetic syndromes in a child affecting communication, and parents who did not complete a majority (at least 4 out of 7) of the daily diary entries.

### 2.2. Parental CAM Attitudes

Parents completed an adapted version of the CAM questionnaire [19], in which the original questionnaire phrasing of “young adults” was replaced with “people.” To assess overall CAM attitudes, parents indicated their degree of agreement with nine statements, which was scored on a 5-point Likert scale ranging from 1 (“strongly disagree”) to 5 (“strongly agree”). Example items included: “There are less side effects when taking natural remedies,” and “CAM involves natural plant formulas which are more healthy than taking drugs given by the medical doctor.” Items were averaged to yield a composite score for each parent, with higher scores indicating more positive CAM attitudes. Internal consistency for the CAM attitudes scale in this study was high (Cronbach’s α = 0.92).

### 2.3. Child Pain Intensity 

In the 7 days immediately following surgery, parents used a daily diary to report their child’s minimum and maximum pain intensity. For children under the age of 6 (n = 5), the FLACC (face, legs, activity, cry, and consolability) pain scale [20] was used. This scale is a behavioral assessment of pain intensity, on which the five behaviors are each rated on a 0 to 2 scale. Items were summed yielding a 0–10 pain intensity score. For children over the age of 6 (n = 28), a numeric pain intensity rating scale was used, ranging from 0 (no pain) to 10 (worst pain possible). To account for potential differences in measurement between these two scales, pain ratings were transformed to z-scores based on each measure.

For data analysis, children’s pain intensity was modeled at both the within-person level and between-person level. The person-centered, within-person component (daily pain intensity) indexed a child’s average daily pain intensity relative to the mean of his/her postoperative pain across the 7-day period. For example, a child with a negative value for daily pain intensity meant that they experienced less pain that day than their average across the diary period. The between-person, sample-centered component (average postoperative pain) captured a child’s mean postoperative pain intensity relative to the mean of the entire cohort.

### 2.4. Clinical Outcomes

Our primary outcome of interest during the post-tonsillectomy recovery course was medication administration. Each day, parents recorded the time (to the nearest hour) and medication (acetaminophen, ibuprofen, or oxycodone) administered. A daily sum was computed for each medication to indicate the number of daily doses administered. Further, a binary variable was coded (0/1) to construct two groups: (0) parents who did not administer any opioid doses over the 7-day period and (1) parents who administered at least one opioid dose over the 7-day period.

### 2.5. Statistical Analysis

Initial descriptive analyses used t-tests to evaluate the differences between categorical variables (sex, any opioid use) on continuous variables (average postoperative pain intensity). Mann–Whitney U tests were used to evaluate total analgesic use across the 7-day diary period (acetaminophen, ibuprofen, and oxycodone were evaluated separately) by patient sex. The relationships between patient age and key postoperative variables were evaluated by Pearson’s r for scale data (average postoperative pain intensity and parental CAM attitudes) and Spearman’s rho for count data (acetaminophen, ibuprofen, and oxycodone doses).

To evaluate both between-person predictors (patient age, parental CAM attitudes and postoperative pain intensity) and within-person predictors (daily pain intensity) of daily medication use over the 7-day postoperative period, generalized linear mixed-effects models (GLMM) with a Poisson distribution were estimated with the lme4 package in R 3.4 [21]. A Poisson distribution was chosen because medication administration data best fits with a count data model (i.e., counts are not normally distributed). GLMM analyses were modeled with diary days clustered within each participant. Three GLMM models were estimated for each of the three outcomes: (1) number of oxycodone doses, (2) number of acetaminophen doses, and (3) number of ibuprofen doses. The models included participant as a random effect, daily pain intensity as a within-person fixed effect, and average postoperative pain and parental CAM attitudes as between-person fixed effects.

Analysis was performed using Stata 16 (StataCorp, College Station, TX, USA), R 3.4 (R Foundation for Statistical Computing, Vienna, Austria), and SPSS (IBM, Armonk, NY, USA), with *p*-values ≤ 0.05 considered statistically significant. As the present manuscript represents a preliminary report based on a secondary analysis of a dataset, an a priori power analysis was not conducted. Instead, 95% CIs are provided for primary findings.

## 3. Results

### 3.1. Participants

In total, 59 parents completed the CAM questionnaire and enrolled in the study, of which 34 (58%) completed valid diary entries for at least 4 of the 7 days. Diary completers and non-completers did not differ with regard to patient age, sex, or parental CAM attitudes. One patient was further excluded due to multiple postoperative ED visits receiving intravenous opioid analgesics, ultimately resulting in 33 patients being included in our study cohort. Of the 33 total patients, 17 (51%) were male, and average patient age was 8.5 years (SD = 2.7). Surveys were primarily completed by mothers (n = 28, 85%). Primary indications for tonsillectomy included obstructive sleep apnea (n = 16, 49%), snoring/sleep-disordered breathing (n = 6, 18%), adenotonsillar hypertrophy (n = 4, 12%), and recurrent pharyngitis (n = 3, 9%; Table 1).

### 3.2. Post-Tonsillectomy Home Analgesic Use

Parents completed a median of 6 diary days (IQR: 4–7), resulting in 189/231 diary assessment data available (82%). In the 7 days post-tonsillectomy, all parents reported administering acetaminophen and ibuprofen, with a median total of 14 acetaminophen doses (IQR: 12.0–18.5) and 15 ibuprofen doses (IQR: 11.0–18.5), while 19 parents (58%) administered oxycodone, with a median of one total dose (IQR: 0–2, range: 0–6). Children who received at least one opioid dose compared to those who did not displayed significantly higher average postoperative pain (t(31) = 2.07, *p* = 0.047; z-score mean = 0.21, SD = 0.70 vs. mean = −0.30, SD = 0.70, respectively). Patient age and sex were not associated with the number of administered doses of acetaminophen, ibuprofen, or oxycodone, the child’s average postoperative pain, or parental CAM attitudes (all *p*-values > 0.05).

**Figure 1:**
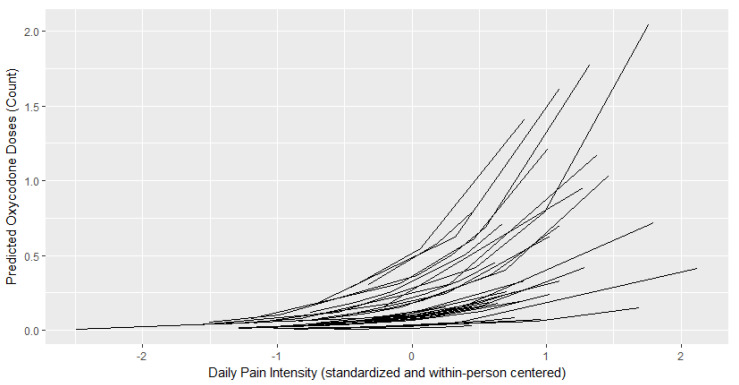
Within-person relationship between a child’s daily pain intensity and predicted oxycodone doses administered. Daily pain intensity is within-person centered where a higher score indicates greater pain intensity on a given day compared to a child’s average across the postoperative period.

**Figure 2:**
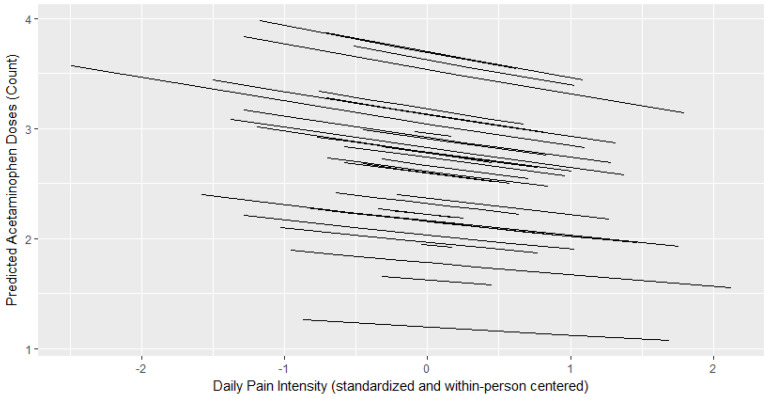
Within-person relationship between a child’s daily pain intensity and predicted acetaminophen doses administered. Daily pain intensity is within-person centered where a higher score indicates greater pain intensity on a given day compared to a child’s average across the postoperative period.

**Figure 3:**
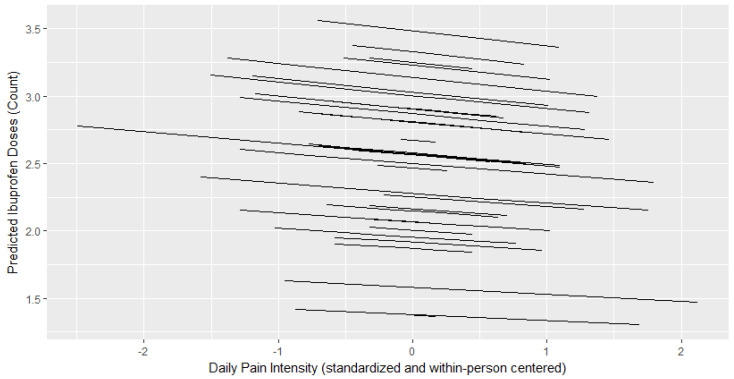
Within-person relationship between a child’s daily pain intensity and predicted ibuprofen doses administered. Daily Pain Intensity is within-person centered where a higher score indicates greater pain intensity on a given day compared to a child’s average across the postoperative period.

Table 2 presents results from GLMMs with a Poisson distribution. Figure 1, Figure 2 and Figure 3 depict the relationship between the child’s daily pain intensity and predicted number of oxycodone, acetaminophen, or ibuprofen doses administered, respectively. The child’s daily pain intensity (within-person predictor) was positively associated with the daily number of administered oxycodone doses (b = 1.23, *p* < 0.001), but not with daily doses of acetaminophen or ibuprofen. This means that more oxycodone doses were administered on a given day if the child was experiencing more pain than their mean daily pain level. The child’s average postoperative pain (between-person predictor) was positively associated with the number of administered ibuprofen (b = 0.244, *p* = 0.001) and oxycodone doses (b = 0.766, *p* = 0.008) across the postoperative period, but not with acetaminophen.

**Figure 4:**
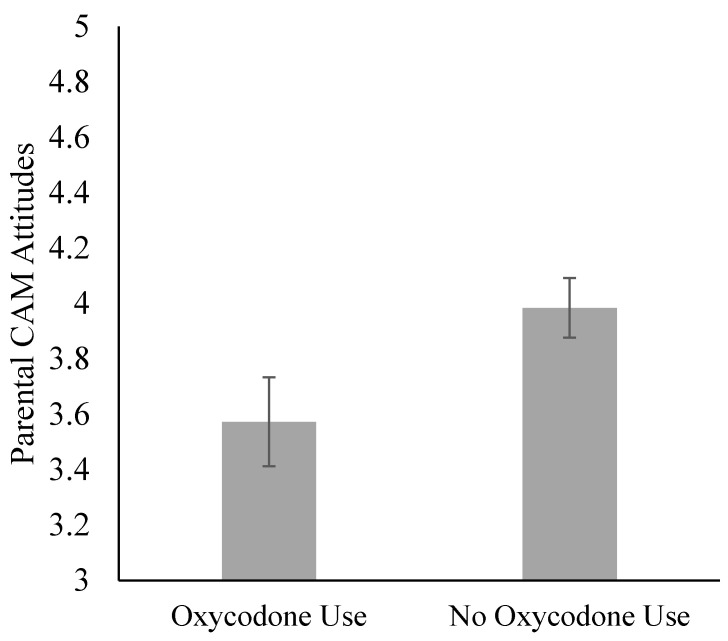
Parental CAM attitudes by oxycodone use during the 7-day postoperative period. Groups differ at the *p* < 0.05 level. Oxycodone use refers to administering at least one dose of oxycodone during the daily diary period.

Parental CAM Attitudes were associated with the likelihood of administering oxycodone post-tonsillectomy, as well as with the number of doses given. Parents who did not administer any oxycodone, when compared to parents who administered at least one dose, reported significantly more positive CAM attitudes, mean = 3.98 (SD: 0.60) vs. mean = 3.57 (SD: 0.47), respectively (t(31) = 2.21, *p* = 0.04, Cohen’s d = 0.76; Figure 4). More positive CAM attitudes predicted fewer doses of administered oxycodone (b = −1.192, *p* = 0.004), above and beyond the medication variations that could be accounted for by daily pain intensity and average postoperative pain.

## 4. Discussion

The present study makes two important contributions to the understanding of pediatric post-tonsillectomy analgesic administration, using a prospective electronic diary to capture both within- and between-person factors. First, parents most often administered opioid analgesics in a rescue context on days when their child’s pain was perceived as greater than average. This same pattern was not observed for the scheduled OTC medications (acetaminophen and ibuprofen). Second, parents with more favorable CAM attitudes gave fewer doses of oxycodone, even when accounting for the impact of individual differences in their child’s pain intensity.

Parental decision-making regarding medication administration was informed by both the parent’s assessment of their child’s pain and their own preferences for natural treatments. Prior studies have proposed that caregiver methods of pain assessment and negative attitudes regarding pain medications influence the pain treatment children receive following surgery [22], and our present study evaluates these associations in a prospective manner. Recent clinical guidelines for pediatric tonsillectomy have emphasized the importance of proper pain education between providers and parents [5,6], encouraging pain management techniques, such as adequate use of preventative (i.e., scheduled) analgesics and a standardized way to communicate about pain [23]. Parents in the present study appeared to follow this advice, using OTC analgesics prophylactically and opioids in response to experienced pain fluctuations. The number of ibuprofen doses administered was positively associated with the child’s average postoperative pain, suggesting that signs of more intense pain during the recovery period may prompt parents to give a longer course or more-frequently-scheduled doses of prophylactic analgesics. Additional education from providers on assessing pediatric pain and appropriate times to administer an opioid could improve parental abilities to provide optimal analgesia, avoiding both undertreatment and overtreatment.

Both the child’s daily pain intensity and parental CAM attitudes independently predicted the number of post-tonsillectomy opioid doses given. Thus, even parents with strong preferences for CAM interventions may choose to administer opioids if their child has significant amounts of pain. Although this threshold will vary across individuals, our study offers some insight into the influences guiding parental approaches to pediatric postoperative pain management. Currently, post-tonsillectomy CAM interventions for pain have limited available evidence [24]. Parents tend to use non-pharmacological strategies such as distraction and deep breathing in the home setting to manage children’s pain [16]. Further research is warranted to establish when, how, and which CAM interventions are most effective for pediatric pain management. Incorporating effective CAM interventions into pediatric postsurgical plans, particularly for parents with favorable CAM attitudes, can increase patient comfort while reducing the number of opioid analgesic doses needed.

Our results showed a stronger influence of daily pain intensity compared to average postoperative pain intensity on opioid administration. Prior studies have reported no differences in postoperative pain experiences between those treated only with OTC analgesics and those using additional opioids for breakthrough pain [25,26]. However, it is important to note that these studies relied on group averages, and thus do not capture individual daily variabilities in pain. Statistical techniques capturing individual differences should be applied to a larger sample size to improve understanding of the instances and patients for which opioids would be helpful for managing pain and improving functioning.

Our study findings should be interpreted in the context of several limitations, including the single institution setting, small sample size, and the number of missing diary entry datapoints (20%). In addition, our cohort had a wide age range (5–17 years) and a different pain assessment tool was used for children under 6 years old (n = 5). However, the generalized mixed-modeling approach is robust for accounting for missing data, and the diary entries utilized numerous individual observations to increase our study’s power. The daily diary structure also allowed us to capture the relationship between experienced pain and administered analgesics on an individual basis to more precisely capture the fluctuating nature of acute postoperative pain and analgesic use. Finally, while our institution typically prescribes three days of postoperative liquid oxycodone for children over 5 years old, policies and treatment patterns may differ elsewhere. The present study should be viewed as a preliminary report in a small sample size and needs to be replicated on a larger scale in a sample with greater variability in opioid use. Future efforts aimed at improving participant retention (as 44% of our enrolled 59 participants did not complete diary entries) could utilize daily scheduled phone calls, fewer diary items, or financial incentives.

This study did not systematically capture the use of a range of CAM interventions for post-tonsillectomy pain on the daily diary. Therefore, the relationship between parental CAM attitudes and use of such interventions in practice is unknown and a goal of current research. Additionally, future electronic diary research could seek to quantify the extent to which medication administration decreases the level of pain experienced. Because our assessments occurred daily, we were unable to evaluate the in-moment responsiveness to medications. Since pediatric pain has been historically undertreated [27,28], it is also important to assess whether avoiding opioid analgesics may lead to greater healthcare use (e.g., physician calls, ED visits) for postoperative pain management, or any functional impairments in children.

## 5. Conclusions

Parents are the gatekeepers of home analgesic use following pediatric surgery. Parental attitudes and beliefs surrounding pain interventions, as well as their ability to assess their child’s pain, have significant implications for postoperative pain management. Both the child’s daily pain intensity and the parent’s preferences for CAM interventions were associated with the number of opioid doses administered following tonsillectomy. Further characterization of factors influencing individual differences in analgesic administration can inform future clinical interventions, such as a precision-medicine assessment questionnaire, to improve discharge planning and parental education for managing postoperative pain.

## Figures and Tables

**Table 1:** Patient characteristics.

Sample Characteristics	Pediatric Patients (n = 33)
Patient Demographics	
Age (years), mean (SD)	8.5 (2.7)
Sex, n (%)	
Female	16 (49%)
Male	17 (51%)
BMI, mean (SD)	18.8 (5.1)
Indication for surgery	
Obstructive sleep apnea	16 (49%)
Adenotonsillar hypertrophy	4 (12%)
Snoring/sleep-disordered breathing	6 (18%)
Recurrent pharyngitis	3 (9%)
Other	4 (12%)
Post-Tonsillectomy	
Diary days completed, median (IQR)	6 (4–7)
Acetaminophen total doses, median (IQR)	14 (12–18)
Ibuprofen total doses, median (IQR)	15 (11–18)
Oxycodone total doses, median (IQR)	1 (0–2)

Note: IQR = interquartile range; SD = standard deviation. Because administered medication doses are count data, medians were chosen as the best representation of central tendency.

**Table 2:** Fixed effects from generalized linear mixed-effects models (GLMM) analyses modeling between-person and within-person predictors of medication administration.

Variable	b	95% CI	SE	z	*p*
Model 1—Number of Oxycodone Doses					
Intercept	2.241	−0.47–5.448	1.473	1.521	0.128
Daily pain intensity	1.23	0.790–1.696	0.230	5.348	<0.001
Average postoperative pain	0.766	0.208–1.402	0.291	2.632	0.008
CAM attitudes	−1.192	−2.128–-0.406	0.419	−2.848	0.004
Model 2—Number of Acetaminophen Doses					
Intercept	1.163	0.212–2.111	0.464	2.504	0.012
Daily pain intensity	−0.065	−0.191–0.062	0.064	−1.009	0.313
Average postoperative pain	0.184	−0.007–0.382	0.0953	1.931	0.054
CAM attitudes	−0.062	−0.317–0.187	0.123	−0.507	0.612
Model 3—Number of Ibuprofen Doses					
Intercept	1.631	0.826–2.455	0.395	4.151	<0.001
Daily pain intensity	−0.032	−0.161–0.097	0.066	−0.4932	0.623
Average postoperative pain	0.244	0.081–0417	0.082	3.000	0.003
CAM attitudes	−0.197	−0.421–0.015	0.106	−1.861	0.063

Note: CAM = complementary and alternative medicine.
